# Complete genome of a rare *Salmonella enterica* subsp. *enterica* serovar Hessarek from human stool

**DOI:** 10.1128/mra.00009-24

**Published:** 2024-08-20

**Authors:** Caitlin A. Selway, Jacob P. May, Shenoi M. A. Goonetilleke, Helen Hocking, Mark Turra, Lex E. X. Leong

**Affiliations:** 1 Microbiology and Infectious Diseases, Public Health Laboratory, SA Pathology, Adelaide, South Australia, Australia; 2 Salmonella Reference Laboratory, SA Pathology, Adelaide, South Australia, Australia; 3 UniSA Clinical & Health Sciences, University of South Australia, Adelaide, South Australia, Australia; University of Maryland School of Medicine, Baltimore, Maryland, USA

**Keywords:** *Salmonella enterica*, food outbreak, human infection, serovar Hessarek

## Abstract

We present a complete genome of *Salmonella enterica* subsp. *enterica* serovar Hessarek isolated from a human stool from an outbreak linked to egg consumption in South Australia. Orientation of the *rrn* operon and characteristics of the *Salmonella* virulence plasmid indicates that this serovar is virulent toward humans and birds.

## ANNOUNCEMENT

*Salmonella enterica* is a common pathogen that infects the gastrointestinal tract of people and animals. *S. enterica* consists of more than 2,500 serovars, and these serovars can be specific to hosts and their environments. *S*. Hessarek—an uncommon non-typhoidal serovar—was previously thought to have high host specificity toward birds but has been shown to also infect humans and other animals through consumption of contaminated the bird itself, eggs, or egg products (1, 2). In 2017–2018, 25 South Australian cases of *S*. Hessarek gastroenteritis instigated an investigation with 96% of cases having consumed eggs and 68% having consumed one specific brand of eggs (3).

An isolate from human feces as part of the 2017–2018 protracted outbreak (3) was plated on XLD Agar (Thermo Fisher) and cultured overnight at 37°C, where a single *S*. Hessarek colony was picked for whole genome sequencing. Genomic DNA was extracted using the QIASymphony DSP Virus/Pathogen kit following manufacturer instructions (QIAGEN). DNA libraries were prepared for Illumina NextSeq using a 2 × 150 bp kit (Nextera-XT for short-reads) and Oxford Nanopore Technologies MinION Mk1c (Ligation Sequencing Kit SQK-LSK109 for long-reads, without shearing, nor size selection). Short- and long-read sequences were demultiplexed and base-called with bcl2fastq (v2.19.0.316) and dorado (v0.3.4, model: dna_r9.4.1_e8_sup@v3.3), respectively. Both sets of sequences were used as input in *Unicycler* (v0.5.0) (4) for *de novo* genome assembly, whereby *Unicycler* trims overlaps and rotates the start position to *dnaA*. Gene annotation, virulence factors, and plasmid identification were predicted with *Prokka* (v1.14.6) (5) and *ABRicate* (https://github.com/tseemann/abricate; v1.0.1) with VFDB (2023-Oct-6) (6) and plasmidfinder (2023-Oct-6) (7) databases. The direction of the rrn gene arrangement was visually inspected using *Proksee* (https://proksee.ca/). All software used default parameters.

**Fig 1:**
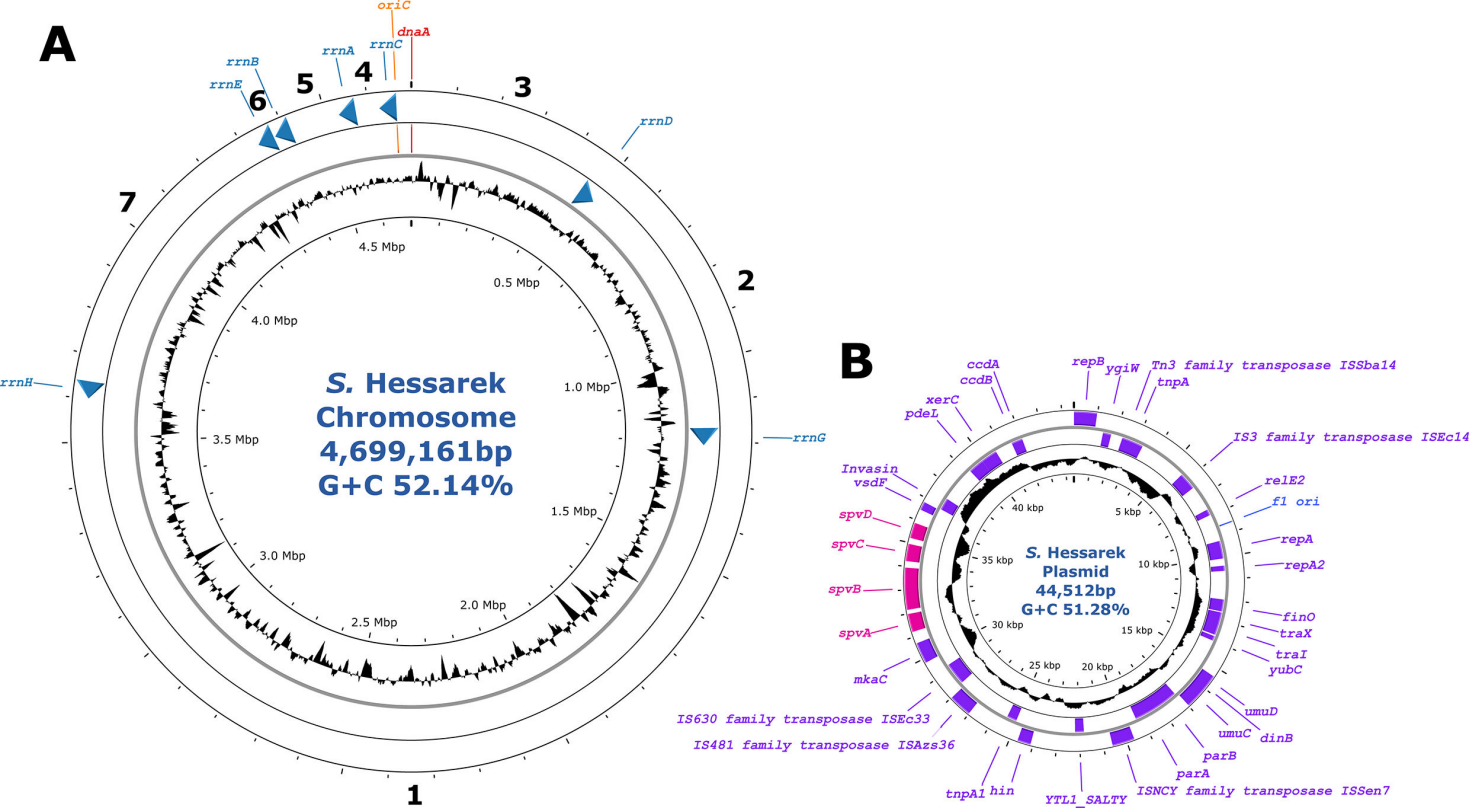
Genomic features of the circularised chromosome and plasmid of *Salmonella* Hessarek. (**A**) Chromosome of *S*. Hessarek including the ribosomal RNA operon genes and their corresponding arrangement. (**B**) Plasmid of *S*. Hessarek including all genes detected using Prokka and highlighting the *spv*ABCD operon in pink.

A total number of 4,921,862 short-read sequences (total yield = 706,844,377 bp; Q-score = 33.1) and 67,594 long-read sequences (total yield = 135,984,456 bp; average length = 2,011 bp; N50 = 4,432 bp; Q-score = 24.6) were used to assemble the *S*. Hessarek genome. The assembled genome consisted of a closed, circular chromosome of 4,699,161 bp with a G+C content of 52.14%, and a single 44,512 bp circularised plasmid (Fig. 1). Through pubMLST, the genome was assigned as sequence type (ST)-255. A total of 4,576 genes were identified within the genome, including 4,471 genes in the coding region, 22 rRNAs, 82 tRNAs, and one tmRNA. Generally, the arrangement of the ribosomal RNA operon (*rrn*) in the chromosome is associated with host specificity. In this genome, the *rrn* operon was arranged as the conserved type “1234567” (Fig. 1) and is associated with broad host specificity (8).

The plasmid was identified as a *Salmonella* virulence plasmid (pSV) due to the presence of the *spv*ABCD operon (Fig. 1) and contained the IncFIB:IncFII replicon. Furthermore, proteins encoded by this operon (i.e., SpvB, SpvC, and SpvD) are translocated into host cells by the Type III secretion system (T3SS), which suppresses host innate immune responses during infection (9). Overall, the arrangement of the *rrn* operon in the chromosome and the T3SS within the plasmid demonstrates that this strain of *S*. Hessarek has broad specificity and the potential to cause virulence in both birds and humans, which contributes to outbreaks from egg consumption in Australia.

## Data Availability

Both Illumina and Oxford Nanopore Technologies raw sequences have been deposited to Sequence Read Archive under the accession numbers SRR27792664 and SRR27792665, respectively. The complete *S*. Hessarek genome sequence has been deposited in the NCBI Genome under the BioSample accession number SAMN38843928.
